# Development of an intervention to incentivize secure firearm storage among veterans at risk for suicide

**DOI:** 10.1186/s40621-026-00674-5

**Published:** 2026-03-28

**Authors:** Gabriela Kattan Khazanov, Gillian Geffen, James McKay, Joseph Simonetti, Ronell Day, Taylor Sczymecki, Samantha Silverman, Gala True

**Affiliations:** 1 Ferkauf Graduate School of Psychology, Yeshiva University, 1165 Morris Park Ave, Bronx, NY 10461 USA; 2 Mental Illness Research Education & Clinical Center, Corporal Michael J Crescenz VA Medical Center, Philadelphia, PA USA; 3 Center of Excellence in Substance Addiction Treatment & Education, Corporal Michael J Crescenz VA Medical Center, Philadelphia, PA USA; 4 VA Rocky Mountain Mental Illness Research, Education, and Clinical Center for Suicide Prevention, Aurora, CO USA; 5 Firearm Injury Prevention Initiative, University of Colorado Anschutz School of Medicine, Denver, CO USA; 6 Southeast Louisiana Veterans Health Care System, New Orleans, LA USA; 7 Corporal Michael J Crescenz VA Medical Center, Philadelphia, PA USA; 8 Section of Community and Population Medicine, Department of Medicine, Louisiana State University Health Sciences Center, New Orleans, LA USA

**Keywords:** Firearms, Incentives, Veterans, Suicide, Community-engaged

## Abstract

**Background:**

Firearm injuries account for 74% of veteran suicides. During lethal means counseling (LMC), clinicians discuss with patients their access to lethal means like firearms and collaboratively develop a plan to reduce this access. However, many patients do not go on to change their firearm storage practices. While financial and social incentives motivate health behavior change, they have not been leveraged to promote secure firearm storage. We used community-engaged methods to develop an intervention offering incentives to encourage secure firearm storage among veterans at elevated risk for suicide.

**Methods:**

We conducted qualitative interviews with 20 veteran firearm owners with recent suicidal ideation (equally split among the Philadelphia and New Orleans regions and among rural and urban-residing veterans) and 10 VA clinicians and administrators with expertise in veteran suicide prevention. We also convened an advisory board with veteran firearm owners and VA clinicians/administrators. Interviews were analyzed using rapid qualitative methods and findings were presented to the advisory board, which employed online modified Delphi procedures to reach consensus on the final intervention features.

**Results:**

Veterans and clinicians were enthusiastic about the potential for incentives to promote secure firearm storage. The final intervention will include two monetary and one social incentive. First, $50 will be offered to veterans who attend a follow-up session to update the clinician about their secure storage plan and a bonus $50 will be offered to veterans who provide verification of secure firearm storage (e.g., a picture or receipt from a recently purchased locking device). The social incentive will include written testimonials from veterans who chose to store their firearms more securely in the context of suicide risk. Incentives will be offered for any meaningful increase in veterans’ secure storage practices using a person-centered framework (e.g., using a locking device, storing an unloaded firearm out of reach).

**Conclusions:**

Using a collaborative and iterative approach, stakeholders developed an intervention leveraging financial and social incentives to encourage secure firearm storage. Study findings have implications for LMC more broadly, including development of acceptable methods of verifying secure firearm storage and identifying ongoing monitoring of firearm storage as key to improving outcomes.

**Supplementary Information:**

The online version contains supplementary material available at 10.1186/s40621-026-00674-5.

## Introduction

Firearm injuries account for 52% of suicides among the general population in the US, and for 74% of suicides among military veterans [1]. In lethal means counseling (LMC), an evidence-informed intervention, clinicians discuss with patients their access to lethal means like firearms and develop a plan to limit this access, typically by storing firearms or other means outside of the home or more securely (e.g., locked, unloaded, separately from ammunition) in the home [2]. LMC can be delivered as a standalone intervention, but is also incorporated into Suicide Safety Planning, a brief intervention in which patients identify coping and safety strategies for use prior to and during suicidal crises [3, 4]. Recent studies demonstrate that LMC delivered as part of Safety Planning was completed by 51% of veterans at elevated risk for suicide across Veterans Health Administration (VHA) settings and firearm-related safety interventions were delivered to approximately 80% of at-risk veterans with documented firearm access evaluated in acute and emergency care settings [5, 6]. While the effect of stand-alone LMC on rates of suicide is unknown, Safety Planning with follow-up has been shown to reduce suicidal behavior and higher quality Safety Plans have been associated with decreased suicidal behavior among veterans in VHA [7–9]. To help identify veterans at risk for suicide, VHA employs both clinician assessment, including identifying potential means for suicide, and a suicide risk prediction algorithm based on electronic health record data [5, 10].

Responses to LMC vary widely, however, with most studies finding improvements in storage behaviors that are not universal and limited quality of evidence, especially as it relates to veterans in VHA [11]. Research on health behaviors has demonstrated that financial and social incentives help motivate behavior change to align more closely with individuals’ goals, such as receiving vaccinations, exercising, and maintaining abstinence from substances [12–14]. While prior studies have shown that clinicians may have concerns about the potential for incentives to negatively impact therapeutic relationships, their enthusiasm for incentive-based interventions tends to increase with training and practical experience delivering them [15–17].

Studies conducted by our team and others have found that patients consistently identify financial barriers to secure firearm storage (e.g., high cost of valued storage devices like biometric lockboxes, hesitation to use low-cost devices like cable locks due to perceptions that they are inconvenient or insecure), note that they would be more likely to store their firearms securely if the costs of storage were defrayed, and find the idea of providing financial incentives to encourage secure firearm storage acceptable [18–20]. Additionally, firearm owners’ perceptions of social support from close others and norms indicating preferences for secure firearm storage in their communities increase the likelihood of following up on secure storage recommendations [21–23]. While some studies have provided no-cost storage devices, typically cable locks, to participants who requested them [24–26], interventions leveraging financial or social incentives to encourage patients to follow through on plans to securely store their firearms have not yet been developed or tested. Importantly, we do not yet have data demonstrating that incentives improve secure firearm storage or decrease suicidal behavior.

In the present study, we aimed to develop an acceptable and feasible intervention offering financial and/or social incentives to encourage secure firearm storage among veterans at elevated risk for suicide, as determined by the widely used Columbia Suicide Severity Rating Scale Screener [27, 28]. Given the sensitivity of conversations about firearms as well as the use of incentives to encourage behavior change, we employed community-engaged methods to shape the intervention around the concerns and perspectives of key stakeholders [21, 29–31]. These stakeholders included veteran firearm owners at elevated risk for suicide and VA clinicians and administrators. We explored the possibility of using three potential monetary or social incentives based on the types of incentives found to be most effective in previous research (see Supplemental Materials for details). For monetary incentives, we considered providing a cash voucher, the opportunity to win money in a lottery, and a cash voucher with loss framing (i.e., framed as already having been earned but at risk of being lost without confirmation of secure storage to activate loss aversion; [32–34]). For social incentives, we considered interventions that capitalized on social support, perceptions of social norms, and social commitment (i.e., stating your intention to complete a task; [14, 21, 35–37].

As research showing that rates of firearm ownership, reasons for ownership, and attitudes towards firearms and secure storage differ in rural versus urban settings, we conducted a two-site study in Philadelphia and New Orleans that provided access to veterans in both urban and rural settings [38–41]. We also endeavored to include female as well as male veterans and those identifying as racial or ethnic minorities because access to firearms and storage practices, as well suicide rates, differ based on these characteristics [42–45]. White veterans report greater access to firearms, and white and American Indian/Alaska Native veterans die by suicide at higher rates than those of other races [44, 45]. While male veterans report greater access to firearms and higher rates of firearm suicide than female veterans, both firearm access and suicide are increasing among female veterans [42, 44, 45]. In this paper, we describe the methods we used to develop an incentive-based intervention promoting secure firearm storage.

## Methods

### Study team

The study team included two PhD trained female researchers employed by the VA, two female masters level research coordinators, one male veteran VA community engagement and partnership coordinator, and one male veteran in training to become a mental health clinician; both veterans use the VA for healthcare. Two team members were based in Philadelphia and the rest were based in New Orleans. Study team members identified as Black American (*n* = 1), White (*n* = 3), and Latine (*n* = 2), and ranged in age from mid-20s to mid-50s. At the time of the study, two team members were firearm owners.

## Participants

### Qualitative interviews–veterans

We included veterans with lived experience of suicidal ideation and firearm ownership. We used electronic health records to recruit veterans with self-reported suicidal ideation over the previous six months, based on the Columbia Suicide Severity Rating Scale (C-SSRS) Screener [27, 28]. The C-SSRS Screener is a widely used, reliable and valid clinician-administered screener for suicidal ideation and behavior that must be administered to veterans at least annually [27, 28]. Veterans were also required to have reported access to firearms either on the Suicide Safety Plan or the Comprehensive Suicide Risk Evaluation (CSRE), a VA-specific clinical tool that facilitates collection of patient-reported data on suicide risk and protective factors, including firearm access [44, 46]. Finally, we required that veterans be connected to outpatient mental health services as we intended to recruit veterans engaged in these services for the pilot trial of the intervention. Given differences in firearm access and firearm suicide based on rurality, sex, and race, we aimed to recruit half of the veterans from rural settings and half from urban settings, as well as at least 25% female veterans and those identifying as racial/ethnic minorities [44, 45]. Sociodemographic data, including rurality, was abstracted from electronic health records during the recruitment process and confirmed with veterans during eligibility screening. Rurality is identified in VA health records based on veterans’ rural-urban commuting area codes [47].

### Qualitative interviews–clinicians

We recruited clinicians and administrators working with veterans in outpatient behavioral health settings across both sites. We identified those active in clinical work and administration related to suicide prevention (e.g., frequent administration of Suicide Safety Plans, formulation of local policies and assessments) by contacting each facility’s suicide prevention team members and behavioral health administrators.

### Advisory board

We convened an advisory board that included veterans with lived experience of suicidal ideation (personal or among a close family member or friend, with no timeframe specified) who currently or previously owned firearms. We identified veteran advisory board members through each facility’s suicide prevention community engagement and partnership coordinators and contacts from The Armory Project, a Louisiana-based community-engaged coalition aimed at reducing veteran firearm suicides [48]. Veterans on the advisory board had not previously participated in interviews. As advisory board members were not considered study participants, we did not formally assess them with the C-SSRS but rather relied on their self-reported experiences during discussions with the study team. We recruited clinicians and administrators with roles in veteran suicide prevention and expertise in LMC; most had participated in qualitative interviews and became interested in the study through participating in interviews. Roles were similarly distributed across both sites.

### Qualitative interviews

With approval from The Central IRB of the VHA, we conducted semi-structured qualitative interviews lasting between 30 and 60 min, during which we asked veterans about firearm ownership and current storage practices, including reasons for ownership and prior discussion of storage with VA providers. We also asked about preferences for the intervention, including preferences for types of monetary and social incentives, strategies to provide verification of secure storage, and intervention delivery (e.g., type of clinician, frequency for follow-up). We were careful to frame questions in terms of preferences versus expectations for effectiveness given the limitations of assessing behavioral expectations and their divergence from actual behaviors [49]. Additionally, we inquired how we might tailor the intervention to deal with challenges, such as when patients have access to multiple firearms that should be secured. Full interview guides are provided in the Supplemental Materials and topics are summarized in Table 1. Interviews were transcribed automatically via Microsoft Teams; the study coordinator compared each transcript against the audio to correct and finalize transcripts.

**Table 1:** Summary of data from qualitative interviews

Monetary incentive preference	Veterans (*N* = 20)	Clinicians/administrators (*N* = 10)
	Voucher incentive = 8; Lottery incentive = 3, Loss framing = 7; None = 2	Voucher incentive = 7; Lottery incentive = 1, Loss framing = 2
Additional thoughts about monetary incentives	Many veterans suggested gift cards or discounts specifically for a firearm locking device as opposed to a generic gift card or cash. Concerns arose for veterans in crisis and the risk of not winning the lottery option and what that might do to their mental state. The social support option was valuable, and many participants felt that it should be particularly successful among veterans because of the military culture of honor. Veterans mentioned wanting to encourage internal change over the long term rather than relying on external incentives	Many clinicians expressed concern regarding coercion or providing money in exchange for an outcome desired by the clinician (secure firearm storage). It was suggested that a third party provide the gift card so as not to have any conflict of interest. A confidentiality issue came up for the lottery option and questions arose of how to document this in the electronic health record. Clinicians emphasized the need for adequate training on how to present a monetary incentive so as not to sound insulting or manipulative
Social incentive preference	Social support = 7, Social norm = 5, Social commitment = 5, all = 1, None = 2	Social support = 3, Social norm = 5, Social commitment = 2
Additional thoughts about social incentives	Most participants liked the idea of including family or friends in a secure storage plan, but some expressed concern. Specific concerns include family and friend accountability and responsibility if the veteran attempts suicide, as well as concern for veterans who do not feel safe enough to open up to family or friends, who do not have close family or friends to confide in, or who fear burdening loved ones. Some participants wanted pictures and videos of how other veterans (specifically requesting stories from an individual who served in the same branch as them) securely store firearms and preferred this over hearing secondhand from the clinician, but also recognize privacy concerns with this option. There were mixed feelings towards the social commitment option, where veterans felt that writing is sometimes easier than verbalizing, so it could be a good option. However, in a high-risk situation it could be difficult for a veteran to come up with their own reasons to securely store their firearms	Most clinicians liked the option of including family or friends but had concern for veterans without a support system as well as concerns for relationship dynamics and if involving others would help or harm a veteran in crisis. Clinicians felt that other veteran accounts were valuable and would encourage video accounts (many use written veteran accounts already). Clinicians wondered if a veteran in crisis would be able to write down their own reasons for secure storage. Time constraints for all options were discussed as well as concern for firearm laws interfering with options (for example, legality of asking a family member or friend to hold onto firearm)
Overall incentive preference	Monetary = 6, Social = 14	Monetary = 4, Social = 6
Additional thoughts about incentives	Some participants suggested providing tickets to sporting or concert events, discounts to websites that sell locking devices, lock boxes, etc. Veterans wanted training on secure firearm storage before they transition out of the military. A couple of veterans suggested faith and education either in person or online as grounds for encouraging veterans to securely store their firearms. One veteran suggested a big brother/big sister type of program that would pair veterans at high risk with veterans who have already participated and succeeded with the program.	
Verification preference	Picture = 11, Receipt = 5, Video = 3, Picture or receipt = 1	Picture = 10
General thoughts about incentives	In general, veterans felt that the program was a good idea. Some veterans expressed concern for the veterans actively in crisis and wondered if an incentive during that time would be effective. Some veterans felt that clinicians should encourage internal change so that an incentive, particularly monetary incentive, isn’t necessary. It was suggested to provide some sort of coin or chip indicating successful participation in the program	Clinicians believe incentives and contingency management are productive and reasonable. Some express concern for the power imbalance and fear of coercion between clinicians and veterans. Clinicians felt it is important for veterans with firearms to have and be given options for how to store them appropriately. There was concern for the ability to verify secure storage. The program should frame the idea of storage around the importance of veteran and family safety and any incentives should be accessed immediately
Additional thoughts about verification	There was the question of how to verify if a friend or family member takes a firearm(s) out of the home of the veteran in question. Veterans would like the option to verify with the clinician in real time and even suggested continued proof over time with additional smaller incentives. Taking a picture of the firearm itself could tempt the veteran to use the firearm if they are already at high risk. Veterans were concerned that a picture of a lockbox could come from anywhere and not represent the veteran’s storage practices. Veterans also expressed concern for having picture evidence in their personal files. If the option of a receipt is chosen, it should include the time and date of purchase.	Clinicians stressed the importance of making verification as easy as possible for the veteran. Clinicians do not want the program to require too much evidence from a veteran so as to maintain a trusting relationship. The method of verification should be a collaborative decision and may require as little as a verbal check-in depending on the relationship between veteran and clinician. An app specific to this program was suggested. Clinicians voiced concern because of their risk of legal responsibility if they were to receive images of a firearm.
Preference for member of treatment team	Some participants felt that anyone who interacts with a veteran at the VA should be able to offer this program but there was a strong preference for a member of the veteran’s mental health team who knows the veteran in question already. Peer specialists were also suggested as well as a member of the suicide prevention team	Most clinicians felt that the mental health clinician who has the closest relationship with the veteran should be the one to deliver the intervention. Some clinicians expressed concern over the comfort level of an unknown clinician intervening in a conversation about firearms and secure storage. Peer specialists or suicide prevention coordinators were also suggested. There is concern for clinicians not having the time to deliver the intervention
Encouraging participation	Participants had many ideas of how to encourage participation in the program. Some methods suggested included providing examples of other veterans who participated in program, creating flyers and posters to put up across the VA or at other events, or sending materials, emails, or newsletters to veterans. Participants also suggested clinicians having the attitude of “nothing to lose” when presenting the program to veterans. Another suggestion was for clinicians to bring up the program during intakes and after completing safety plans. It may be helpful to start with the “punchline” (chance to earn money) and to frame as a safety program as opposed to a program to limit access to firearms	Clinicians need training/workshops on how to talk about firearm storage and why it’s important in order to speak with veterans in an effective manner. It may be helpful to promote the program in monthly mental health staff meetings or create a video discussing the program to show to clinicians and on screens throughout the VA. Another way to encourage participation for veterans and clinicians would be for the suicide prevention team to pick one clinician per quarter to be the “Safety Plan Champion” responsible for promoting the program. Finally, clinicians requested making it quick and easy for clinicians to document these conversations in health records
How to manage multiple firearms	Participants believe that all firearms that a veteran has access to should be discussed	Clinicians believe that all firearms that a veteran has access to should be discussed
Method of storage clinicians should encourage	Participants think clinicians should encourage firearm and ammunition to be stored separately. Lockboxes are preferred over trigger locks. Veterans felt clinicians should encourage veterans to store their firearms outside of the home but emphasized the importance of keeping in mind the reason a veteran owns their firearm. If it is for protection, then they may not respond well to requests to remove firearms from the home. If a veteran cannot afford a safe big enough for their firearms, clinicians should encourage them to at least store their ammunition separately and locked up	Clinicians wanted to provide a stratified list of best storage methods with suggestions that vary by risk level of the veteran, with out of home storage being the standard for higher-risk veterans. Clinicians emphasized the importance of keeping in mind a veteran’s reason for owning firearms. Ultimately, anything that adds time and distance between the veteran and firearm should be incentivized
Clinician frequency of follow-up	Some veterans suggested same day follow-up, then weekly, then monthly while others suggested following up at every meeting with a MH clinician. As a veteran improves, participants suggested leaving decisions about frequency of assessment up to the clinician. It was suggested that other generic outreach opportunities (like flu shot reminders) could be used to check in about firearm storage	If a veteran is involved in therapy, then the clinician should be checking in about firearm storage at every session. Clinicians suggested that the frequency of assessment should depend on the veteran’s risk level and that secure storage should be discussed when the suicide prevention team contacts a veteran. Most clinicians expressed concerns regarding the time required to complete follow-ups. Clinicians also suggested providing smaller additional incentives for each follow-up
Tailoring the intervention	In order to tailor the intervention to a veteran’s needs, participants suggested presenting each veteran with multiple acceptable storage options. Participants wanted clinicians to talk to veterans about the incentive program before they are in crisis. It is important not to treat firearms as a taboo subject and to provide information and training on the program to all clinicians, and especially to educate veterans about the statistics on firearm suicides in the US	Clinicians wanted to differentiate the program for veterans who live alone vs. with family, rural vs. urban, and reason for owning firearms. Clinicians highlighted the need to normalize firearm ownership

Monetary incentives included [1] voucher incentive: offering veterans a gift card with a set amount of money for storing their firearms more securely (e.g., $50); [2] lottery incentive: offering veterans the chance to win a larger gift card for storing their firearms more securely (e.g., including them in a lottery with a 25% chance of winning $200); [3] loss framing: presenting veterans with a gift card (for $50, for example) assuming that they will store their firearms more securely, and then not providing the gift card if the veteran does not make this change. Social incentives included [1] social support: clinicians working with veterans to choose a family member or friend they trust to help the veteran store their firearms more securely; [2] social norm: clinicians sharing real examples of other veterans who decided to store their firearms more securely because they had concerns about keeping themselves safe from suicide; [3] social commitment: clinicians asking veterans to write down their reasons for storing their firearms more securely to benefit other veterans in the future

The types of monetary incentives assessed included (Supplemental Materials): [1] *voucher*: offering veterans a gift card with a set amount of money for storing their firearms more securely (e.g., $50) [2], *lottery*: offering veterans the chance to win a larger gift card for storing their firearms more securely (e.g., including them in a lottery with a 25% chance of winning $200) [3], *loss framing*: presenting veterans with a gift card (for $50, for example) assuming that they will store their firearms more securely, and then not providing the gift card if the veteran does not make this change at the subsequent meeting. The voucher and loss framing options differed only in the presentation of the incentive to the veteran, not in the logistics of delivery. The types of social incentives assessed included: [1] *social support*: clinicians working with veterans to choose a family member or friend they trust to help the veteran store their firearms more securely [2], *social norm*: clinicians sharing real examples of other veterans who decided to store their firearms more securely because they had concerns about keeping themselves safe from suicide [3], *social commitment*: clinicians asking veterans to write down their reasons for storing their firearms more securely to benefit other veterans in the future.

Given the need to share interview findings with our advisory board members and to inform intervention development within a short timeframe, we analyzed interviews via rapid qualitative methods using a matrix approach adapted for health services research [50, 51]. Rapid qualitative methods have demonstrated rigor and are well-suited for intervention development and implementation in health care settings [52, 53]. Three of the authors created a summary template draft based on the topics covered in each interview guide and assessed usability by applying it to a subset of the transcripts. We reviewed and resolved any challenges in using the template to summarize transcripts and came to consensus on final templates. Two authors with experience in qualitative coding summarized an equal number of transcripts and transferred summary text and illustrative quotations to a matrix that organized responses across respondents. The authors reviewed each other’s work and resolved disagreements through consensus discussions. The full research team reviewed the final matrix to identify and discuss trends and differences across the data.

### Advisory board

**Fig. 1:**
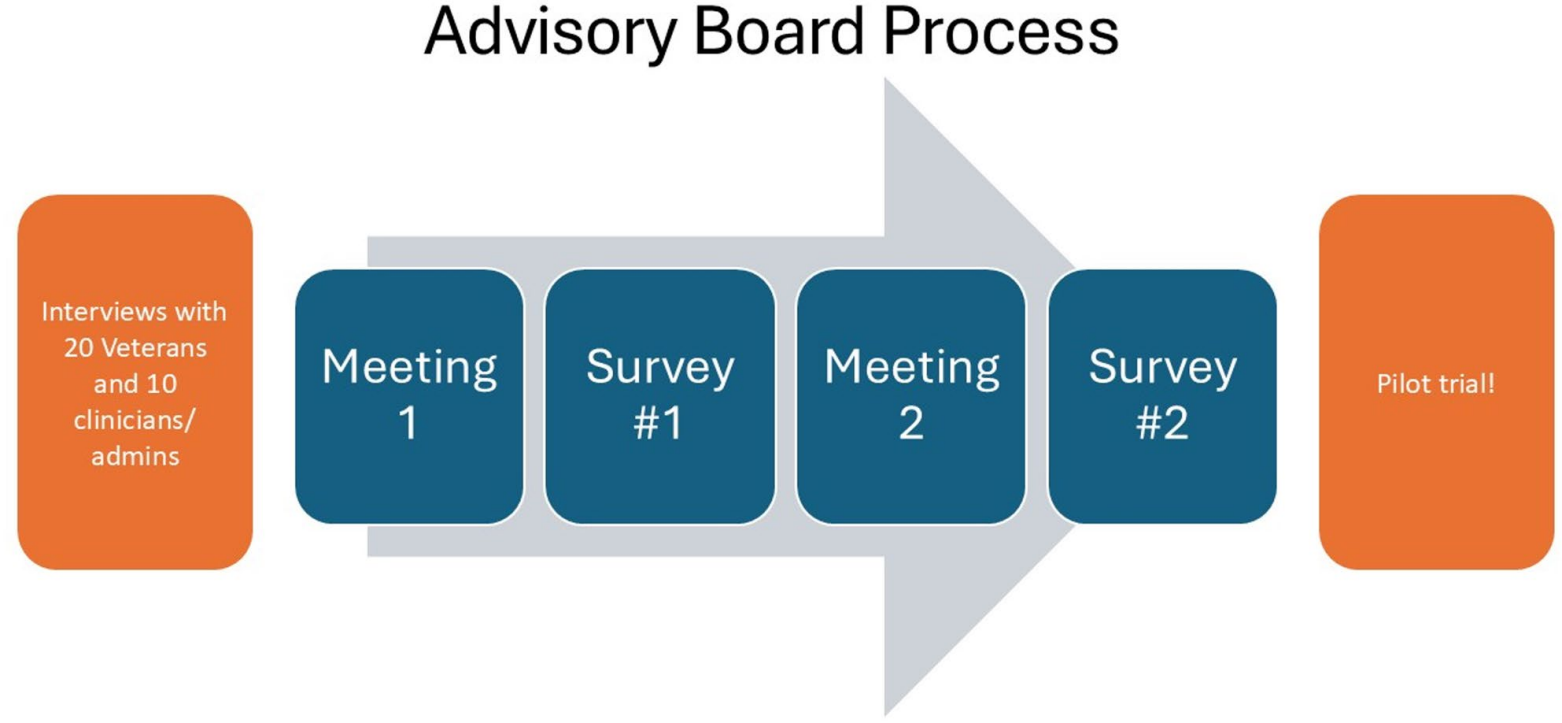
Advisory board online modified Delphi procedures summary

Following the analysis of interviews, we translated key information into graphics and summary points to present to the advisory board (Supplemental Materials). We then met twice with the advisory board and used online modified Delphi procedures to reach consensus on the final features of the intervention (Fig. 1). Following each meeting, board members completed a 5–10 item survey to gather quantitative data on preferences for the intervention (Table 2). Prior to the first official advisory board meeting, we met separately with just veteran, non-clinician members to present background information about the study, including information about suicide prevention, lethal means safety, and the use of incentives to encourage health behaviors, as well as information about the upcoming Delphi procedures.

**Table 2:** Description of advisory board process

	Discussion 1 (*N* = 7; 3 clinicians and 4 Veterans)	Survey 1 (*N* = 12; 6 clinicians and 6 Veterans)	Discussion 2 (*N* = 12; 6 clinicians and 6 Veterans)	Survey 2 (*N* = 8; 3 clinicians and 5 Veterans)	Additional comments from administrators
Discussion points	(1) Reviewed study goals/methods and Aim 1 findings; (2) Reviewed pros/cons of financial incentives options (option 1; incentivizing secure storage, option 2; incentivizing attending follow-up session, option 3; providing some money with no strings attached and incentivizing attendance at a follow-up meeting; option 4; providing a voucher to use for purchasing a locking device - in the future, not possible for pilot trial). (3) Discussed whether we should include a social incentive	(1) Greatest preference for incentivizing follow-up session (option 2), followed by incentivizing secure storage (option 1), then providing a voucher (option 4), and finally providing some money with no strings attached and incentivizing attendance at a follow-up meeting (option 3). (2) Financial incentive amount averaged $50. (3) Preference for enabling veterans to provide verification of secure storage using the most convenient method. (4) Most support for social norm incentive, followed by social support incentive and then social commitment incentive	(1) Reviewed results of survey and discussed option of incentivizing a follow-up session and offering a bonus for verification of secure storage. (2) Discussed preference for clinicians or research team to deliver the intervention, and which veterans to recruit, (3) Discussed process of intervention	(1) Preference for asking clinicians to deliver the intervention versus study staff. (2) How recent should a Safety Plan or evaluation be to contact a veteran about their firearm storage? (3) Responses to firearm storage graphic	OK to incentivize storing outside of the home but avoid making recommendations for out-of-home storage that may run into legal issues
Final takeaways and decisions	Preference for a lower-pressure financial incentive like incentivizing attendance at a follow-up meeting versus secure storage; some concern about asking for verification of secure storage. Support for providing a voucher to minimize inconvenience of purchasing a locking device. Also support for tailoring incentive to veteran and their level of readiness. Overwhelming support for including social norm incentive. Decided to include social incentive and will follow-up with survey about financial incentive	Will include an incentive for attending a follow-up session, and possibly also an incentive for securely storing firearms. Incentive will be around $50. Social incentive will be written or video testimonials from veterans who decided to securely store their firearms (social norm)	Strong support for option of incentivizing follow-up session with a bonus for verifying secure storage; felt that this balanced not pressuring veterans with providing motivation for secure storage. Reaching out to veterans who have recently discussed firearm storage may take less time and be less risky, but there may be a benefit to the study team tracking down non-responsive veterans to ask about secure storage. Having the veteran’s clinician offer an incentive is beneficial because the trusting relationship between clinician and patient is already there, but clinicians are very limited in the time they have and there is a lot of value in the research team helping clinicians follow-up with veterans, especially those who have fallen through the cracks, and offer an additional incentive to encourage secure storage	(1) All but 1 clinician preferred that veterans’ clinicians offer incentives. (2) No clear answer emerged - equal preference for 2 weeks, 6 months, and 1 year, with some support for 3–7 days or 1 month. (3) Approval of firearm storage graphic with no comments for improvement	Instead of only recruiting veterans based on Safety Plans, also recruit Veterans who have recently completed a Comprehensive Suicide Risk Evaluation, which also asks about firearm access. The study is likely only feasible if study staff provide the intervention; training clinicians is likely to be very difficult given their workload

## Results

### Participants

Among veterans who participated in qualitative interviews, 70% were male, 40–50% identified as White, 20–30% as Black, 0–20% as multiracial, and 10–30% as Hispanic/Latine depending on the site (Table 3). Veterans were split equally among the two sites and settings (urban versus rural; 90% of veterans from New Orleans lived in rural settings). We interviewed a total of 10 clinicians and administrators (6 male), with five from each site. Clinicians included five social workers, three psychologists, and two psychiatrists. Among these clinicians, administrative responsibilities included two team leaders, a program manager, mental health assistant chief, and chief of outpatient mental health. One clinician was a member of the facility’s suicide prevention team, a team that reaches out and provides services to veterans at high risk for suicide. Most clinicians had been working at the VA for 5–10 years (*n* = 4), 10–15 years (*n* = 1), or 15–20 years (*n* = 4), with one working for 1–2 years. We did not collect additional demographic details from clinicians and administrators to ensure their confidentiality.

**Table 3:** Demographic characteristics of veterans participating in qualitative interviews by site

Philadelphia	Total	Percent	New Orleans	Total	Percent
*N*	10		*N*	10	
**Gender**			**Gender**		
Male	7	70%	Male	7	70%
Female	3	30%	Female	3	30%
**Race**			**Race**		
Black	2	20%	Black	3	30%
White	5	50%	White	4	40%
Multiracial	2	20%	Multiracial	0	0%
Other	1	10%	Other	3	30%
**Ethnicity**			**Ethnicity**		
Hispanic or Latine	1	10%	Hispanic or Latine	3	30%
Not Hispanic/Latine	9	90%	Not Hispanic/Latine	7	70%
**Age**			**Age**		
18–39	3	30%	18–39	3	30%
40–54	2	20%	40–54	5	50%
55+	5	50%	55+	2	20%
**Setting**			**Setting**		
Urban	9	90%	Urban	1	10%
Rural	1	10%	Rural	9	90%

The advisory board included six veterans (four male; two from rural communities) and six clinicians or administrators (three male). Clinicians and administrators (all of whom were also clinicians) included a team leader, program manager, mental health assistant chief, chief of outpatient mental health, a member of one of the facility’s suicide prevention team, and a clinician without an additional administrative role. Most clinicians and administrators previously participated in qualitative interviews.

### Qualitative interviews

#### Incentive options

Veterans and clinicians expressed strong support for the possibility of offering both monetary and social incentives to encourage secure firearm storage among veterans at risk for suicide (Table 1). Most participants favored straightforward monetary vouchers (voucher incentive; 8 veterans and 7 clinicians) over the chance to win money in a lottery (lottery incentive; 3 veterans and 1 clinician) or framing the incentive as already having been earned but at risk of being lost without confirmation of secure storage (loss framing; 7 veterans and 2 clinicians). Veterans also expressed a preference for gift cards or discounts for firearm locking devices as opposed to cash rewards. Clinicians reported concerns about the ethical implications of potentially coercing veterans to follow clinician advice by offering a monetary incentive.

In terms of social incentives, clinicians favored providing veterans with real examples of other veterans who decided to store their firearms more securely in the context of suicide risk (social norm; 5 clinicians), followed by working with veterans to choose a trusted family member or friend to help them store their firearms securely (social support; 3 clinicians) and then asking veterans to write down their reasons for storing firearms more securely to benefit other veterans in the future (social commitment; 2). Veterans preferred the social support option (*n* = 7) over the social norm (*n* = 5) or social commitment (*n* = 5) options. Both veterans and clinicians stated that while recruiting social support can be very effective, many veterans do not have supportive relationships or do not feel comfortable disclosing their firearm ownership or suicidal ideation to family members or friends.

When asked to compare the different types of incentives, veterans and clinicians expressed more support for social (14 veterans and 6 clinicians) than monetary incentives (6 veterans and 4 clinicians). Concerns were also raised about administering an incentive-based intervention to veterans who may be in crisis and the potential for power imbalances between clinicians and veterans or feelings of coercion among veterans to discourage engagement in the intervention or secure firearm storage more broadly – the importance of increasing internal motivation for secure firearm storage was highlighted.

### Other intervention features

In general, veterans and clinicians preferred flexibility for providing verification of secure storage, with clinicians leaning towards trusting veterans with less or no verification and veterans raising questions about the potential for dishonesty. Both veterans and clinicians also preferred that a clinician who knows the veteran well deliver the intervention given the importance of trust in lethal means safety conversations. A harm-reduction approach, in which veterans are encouraged to store their firearms as securely as they are willing, was supported by both veterans and clinicians. For example, while storage outside of the home is the safest option, veterans would also be incentivized to more securely store their firearms at home (e.g., using a lockbox) if that was the most stringent safety measure they were willing to take. Both groups also underscored a need for continuous monitoring of secure firearm storage, even following the intervention, as well as increased clinician training to encourage clinicians to feel more comfortable and informed when discussing firearms. We did not observe any differences in perspectives among veterans from rural versus urban settings.

### Advisory board processes

#### Incentive options

During the first advisory board meeting (Table 2), board members coalesced around the idea of offering veterans a financial incentive to attend a follow-up meeting with the clinician to provide an update on their firearm storage (seen as a “lower pressure” option) versus offering an incentive for secure storage specifically (seen as a “higher pressure” option). When the option of adding a bonus for verifying secure storage was presented, such that incentives would be offered both for attending a follow-up meeting and for verifying secure storage, the advisory board was enthusiastic about that approach. All advisory board members, including both clinicians and veterans, were also supportive of the social norm incentive – namely, providing veterans with testimonials of other veterans who decided to securely store their firearms. This option was seen as more universally appealing and acceptable for all veterans regardless of their baseline levels of social support. The advisory board decided to prioritize the social the prospect of having to share a video link

### Other intervention features

While advisory board members indicated their interest in veterans’ personal clinicians offering incentives given preexisting trust within this relationship, it was also made clear that clinicians’ time is limited and that clinicians would appreciate the research team following up with veterans themselves instead of adding additional tasks for clinicians to complete. In an additional meeting with four local and national VA administrators, the research team arrived at the conclusion that it would be infeasible to train all behavioral health clinicians to administer the intervention with fidelity and provide it to their patients given their workloads; instead, the research team would offer the intervention and if found to be effective, would subsequently make attempts to have VA clinicians implement it themselves. Alternatively, some clinicians suggested having an external team like each facility’s suicide prevention team, responsible for following up with high-risk veterans, eventually administer the intervention to support clinicians. In addition, despite the advisory board’s preference for providing veterans with a voucher for a lockbox of their choice to minimize the inconvenience of purchasing one (or other items that may encourage secure firearm storage, like security cameras to allow veterans to feel safer in their homes), discussions with VA administrators revealed that this option was not logistically possible for the pilot study being planned. No consensus emerged around the ideal amount of time to follow up with veterans after discussions with their clinicians about secure firearm storage.

### Final intervention features

The final intervention includes two monetary incentives and a social incentive (Table 4). The first monetary incentive ($50) will be offered to attend a follow-up session updating the clinician about the veteran’s secure storage plan and the second monetary incentive ($50, conceptualized as a “bonus”) will be offered for verification of secure storage. The social incentive will include written testimonials from veterans who chose to store their firearms securely in the context of elevated suicide risk. The intervention will be delivered by a clinician on the research team, and verification will be in the form of a picture or receipt from a recently purchased locking device or space in a storage facility or a note from a family member or friend holding on to the patient’s firearms, transferred to the study team via any secure method, or showing the clinician these items during a video call. Clinicians will discuss veterans’ storage practices for all firearms in their homes and will provide incentives for any meaningful increase in veterans’ secure storage practices. Additional details about the intervention are listed in Table 4, and the testimonials we gathered are presented in Table 5.

**Table 4:** Final intervention features

Feature	Description
Monetary incentive	A $50 incentive for attending the follow-up session and updating the clinician about the firearm storage plan (voucher incentive). A $50 bonus incentive for providing verification of secure firearm storage
Additional comments	Configured to minimize clinician concerns about veterans taking offense to the incentive offer or feeling coerced to change firearm storage
Social incentive preference	Written testimonials from other veterans who chose to store their firearm securely in the context of elevated suicide risk (social norm incentive)
Additional features	Clinicians preferred a written list with a variety of different examples that could be offered depending on the circumstance
Additional components related to incentives	Social incentive offered during intervention; monetary incentive offered at follow-up
Verification type	A picture or receipt from a recently purchased device or space in a storage facility, or a note from a family member or friend holding on to the patient’s firearms
Verification transmission	Any secure method of transferring data, including a VA-approved app, encrypted email, electronic health record messaging, and showing a clinician during a telehealth session
Additional thoughts about verification	Clinicians and veterans preferred maximal flexibility
Preference for member of treatment team	Ideally, the veteran’s own clinician or a member of the treatment team. Within the study, a study-based clinician
Encouraging participation	Proactively reaching out to eligible veterans without relying on clinician referral
How to manage multiple firearms	Clinician and patient to discuss using collaborative decision-making; focus on firearm that is most easily accessible and/or the firearm that the veteran thinks of using as a means of suicide
Method of storage clinicians should encourage	Clinician and patient to discuss using collaborative decision-making and a harm-reduction perspective; focus on minimizing access to firearm
Clinician frequency of follow-up	One follow-up session within 1–3 weeks

**Table 5:** Social norm incentive: testimonials from other veterans who chose to store their firearms securely in the context of suicide risk

1. **Veteran A (John)** was in his 60s and owned several firearms that he used primarily for hunting and target practice. He had been successfully treated for lung cancer in his 50s, but recently received news from his doctor that the cancer had returned and spread to other parts of his body. Although John’s doctor told him that he still had options for treatment, John felt depressed and hopeless at the thought of having to go through cancer treatment again and was having thoughts of suicide. He shared this with his wife, who asked him if they could temporarily remove their guns from the house until John felt better. John agreed, and their adult son came to pick up their guns and ammunition to store in his gun cabinet
2. **Veteran B (Maya)** was in her 40s and lived with her teenaged son. She kept a loaded pistol in her bedside table for home defense. Maya had thoughts of suicide over the years but never wanted to leave her son without a mother. She had been going through a particularly hard time due to the death of a close friend and dealing with her son being bullied at school. Her son had also told her that he was feeling depressed and hopeless. Maya decided it would be best if she and her son didn’t have access to a loaded firearm, so she gave her ammunition away to a friend and locked the pistol in a lockbox. She improved her home defense by installing security cameras and an alarm system
3. **Veteran C (Joe)** was in his 30s and prided himself on being prepared for any situation. But after returning home from his second deployment, Joe’s life shifted. When he became a father, he realized the importance of creating a safe environment for his curious, adventurous children. Joe also struggled with moments of anxiety and depression. When considering locking up his firearms, Joe first felt that this might compromise his ability to protect his home. But after researching, he realized there were options that offered both accessibility and security. Joe invested in a high-quality safe, which allowed him quick access in an emergency while ensuring that his firearms were securely stored when not in use. This choice wasn’t just about his family’s safety; it became a part of his healing journey. Joe found that securing his firearms brought him a sense of control and stability, reinforcing his role as a protector in a new, healthier way. Today, he speaks openly with fellow veterans about his decision, encouraging them to prioritize safety without compromising their values. For Joe, securely storing his firearms wasn’t just about meeting a responsibility—it was a way to honor his service and protect the growing legacy of his family
4. **Veteran D (Beth)** was in her 20s and transitioning from the military to civilian life. She was living in her parents’ home and had just broken up with her partner. Beth was trying to get care for her chronic pain but having a hard time getting the medications and treatment she needed. Beth only wanted to store her firearms with someone she trusted, but she didn’t know who would hold on to them for her. She remembered that a local gun shop was run by another veteran, so she stopped by to ask if they would store her firearms. They agreed, and she felt much calmer knowing that her firearms were in good hands. Once Beth started to feel better, she got her firearms back from the gun shop
5. **Veteran E (Sam)**, in his 70s, noticed that he was drinking more and feeling depressed, and his friends and family expressed concerns about his health. He knew that he had to do something to make it through this rough patch but he was too anxious to store his firearms outside of his home. He decided to store them in a safe in his garage to make them more difficult to reach, but still accessible in case he needed them. On the safe, he attached pictures of his kids to remind himself of how much they cared for him. One time Sam considered using his pistol for suicide but walked away with the picture instead

Examples are based on true stories, with details changed to preserve confidentiality

## Discussion

Previous research highlights multiple barriers to secure firearm storage following LMC [11, 18, 21]. While incentives increase behavior change across health domains, they have not yet been applied to promoting secure firearm storage [12–14]. We describe the development of an intervention leveraging financial and social incentives to encourage secure firearm storage among veterans at elevated risk for suicide following LMC. This intervention combines financial incentives in the form of two separate $50 payments for attending a follow-up session and for providing verification of secure firearm storage, along with social incentives in the form of testimonials from veterans who chose to store their firearms more securely in the context of suicide risk. These incentives may help motivate behavior change by enabling veterans to purchase secure storage devices or other safety equipment and to store their firearms in a manner that aligns with their values as firearm owners [18, 21]. By using a community-engaged approach, we designed a financial incentive that both veterans and their clinicians considered acceptable instead of coercive.

This intervention builds on existing initiatives implemented in healthcare settings such as the VA, which distributes cable locks and, more recently, lockboxes at no cost to veterans at elevated risk for suicide who receive a referral from their clinician [44, 54], by examining additional ways in which financial incentives can be leveraged to facilitate secure storage. The current study also complements other interventions being developed that utilize discounts for firearm locking devices or no-cost device provision through retailers [48]. The addition of a social incentive is also novel. While in qualitative interviews veterans indicated a slight preference for the social support versus social norm incentive, all advisory board members, including veterans, decided to prioritize the social norm incentive to avoid excluding veterans who perceive that they do not have someone they could involve in facilitating secure firearm storage. We consulted with stakeholders regarding methods for verifying secure firearm storage and found that pictures or receipts from recently purchased locking devices or storage spaces in a storage facility were considered acceptable forms of verification. Currently, LMC studies rely on self-reported behavior change, but researchers could consider requesting verification of secure firearm storage using these methods to enhance the validity of LMC outcome measures. Implementing verification procedures in clinical practice may present challenges, however, including those related to data privacy and security.

Given that both veterans and clinicians emphasized the importance of ongoing monitoring of firearm storage following LMC, future work should examine how check-ins could be integrated into routine clinical care, including the optimal frequency of monitoring needed to support sustained secure firearm storage. Additionally, both veterans and clinicians emphasized the importance of not coercing veterans into firearm storage practices they view as unacceptable, highlighting the need to approach this and similar interventions within patient-centered and harm reduction frameworks [2, 55].

Importantly, although logistical considerations require the use of cash payments as financial incentives for the pilot study, stakeholders preferred offering funds that could only be used to purchase firearm locking devices or other equipment to help veterans feel safer in their homes (e.g., alarms, lights), which could be accomplished through partnerships with retailers. The fact that veterans consistently preferred incentives that could only be used to purchase locking devices or safety equipment demonstrates their commitment to changing storage behaviors without having to make tradeoffs against other financial needs. Moreover, although both veterans and clinicians preferred that veterans’ own providers deliver this intervention due to the importance of trust in LMC implementation [21, 42, 56], competing demands on VA clinicians’ time required relying on study clinicians to implement the intervention. Importantly, as this intervention is brief and straightforward, there are multiple mechanisms through which it could be implemented into routine clinical practice if shown to be effective. Overall, this intervention holds promise for broader application beyond veterans in VA. Pending evidence of acceptability, feasibility, and effectiveness, similar monetary and social incentive-based approaches could be adapted for other clinical and community settings.

This study should be considered alongside its limitations. First, the intervention was developed using a relatively small sample size for qualitative interviews, and many of the clinicians and administrators serving on the advisory board previously participated in qualitative interviews. Relatedly, qualitative intervention development studies cannot be widely applied out of the setting in which they were developed and rely on self-reported preferences rather than observed behavioral changes. Furthermore, this intervention development study is motivated by research showing the effectiveness of financial and social incentives in promoting behavior change but does not demonstrate that provision of incentives improves secure firearm storage or reduces suicidal behavior. Additionally, logistical constraints limited the types of monetary incentives that were offered for implementation during the pilot study – as the pilot study will be conducted in a healthcare setting, establishing collaborations with external vendors was not feasible. While we included veterans identifying as female and racial or ethnic minorities, our sample size was too small to investigate meaningful differences in preferences or attitudes among these groups. Future studies could examine whether there are differences in preferences for social or financial incentives to motivate firearm storage behavior changes among different subgroups of veterans and other firearm owners.

## Conclusions

As incentives help motivate behavior change, we aimed to leverage incentives to increase secure storage of firearms in the context of suicide risk. By employing stakeholder-engaged methods, we developed an intervention using financial and social incentives to promote secure firearm storage following LMC among veterans at elevated risk for suicide. The intervention, which was approved by both veterans and clinicians on the advisory board, will be pilot tested in VA outpatient behavioral health in both Philadelphia and New Orleans to formally assess its acceptability and feasibility. Further testing with a randomized controlled clinical trial will be necessary to evaluate the intervention’s effectiveness vis-à-vis LMC as typically delivered. Collaborating with external vendors to offer incentives that can be used only to purchase locking devices or other safety equipment would improve the intervention’s acceptability. Study findings – including the development of acceptable methods of verifying secure firearm storage, the identification of ongoing monitoring of firearm storage following LMC as key to improving outcomes, and the process of identifying financial incentives considered to be non-coercive by stakeholders – have implications for lethal means safety interventions across contexts.

## Supplementary Information

Supplementary Material 1

## Data Availability

The dataset of qualitative interviews generated and analyzed during the current study is not available to the public due to concerns about maintaining confidentiality of interviewees. Summaries of data are included in this published article.
