# Supplementary material for: Development of an intervention to incentivize secure firearm storage among veterans at risk for suicide
Source: Inj Epidemiol. 2026 Mar 28;13:33. doi: 10.1186/s40621-026-00674-5 (PMC13151219; doi:10.1186/s40621-026-00674-5)
Supplement: Supplementary file 1 — Supplementary Material 1 [file 40621_2026_674_MOESM1_ESM.docx]

**Supplemental Table 1.** Types of incentives considered for intervention

| Monetary Incentives |  |
| --- | --- |
| Voucher | Offering a gift card with a set amount of money for storing firearms more securely (e.g., $50) |
| Lottery | Offering the chance to win a larger gift card for storing firearms more securely (e.g., a 25% chance of winning $200) |
| Loss framing | Presenting a gift card (e.g., for $50) assuming the patient will store their firearms more securely and then not providing the gift card if this change is not made. |
| Social Incentives |  |
| Social Support | Working with patients to choose a family member/friend they trust to help implement firearm storage changes |
| Social Norms | Sharing real examples of other patients who decided to store their firearms more securely due to concerns about suicide |
| Social commitment | Asking patients to write down their reasons for storing firearms more securely to benefit others in the future |

**Supplemental Figure 1.** Example of presentation of information to advisory board

**
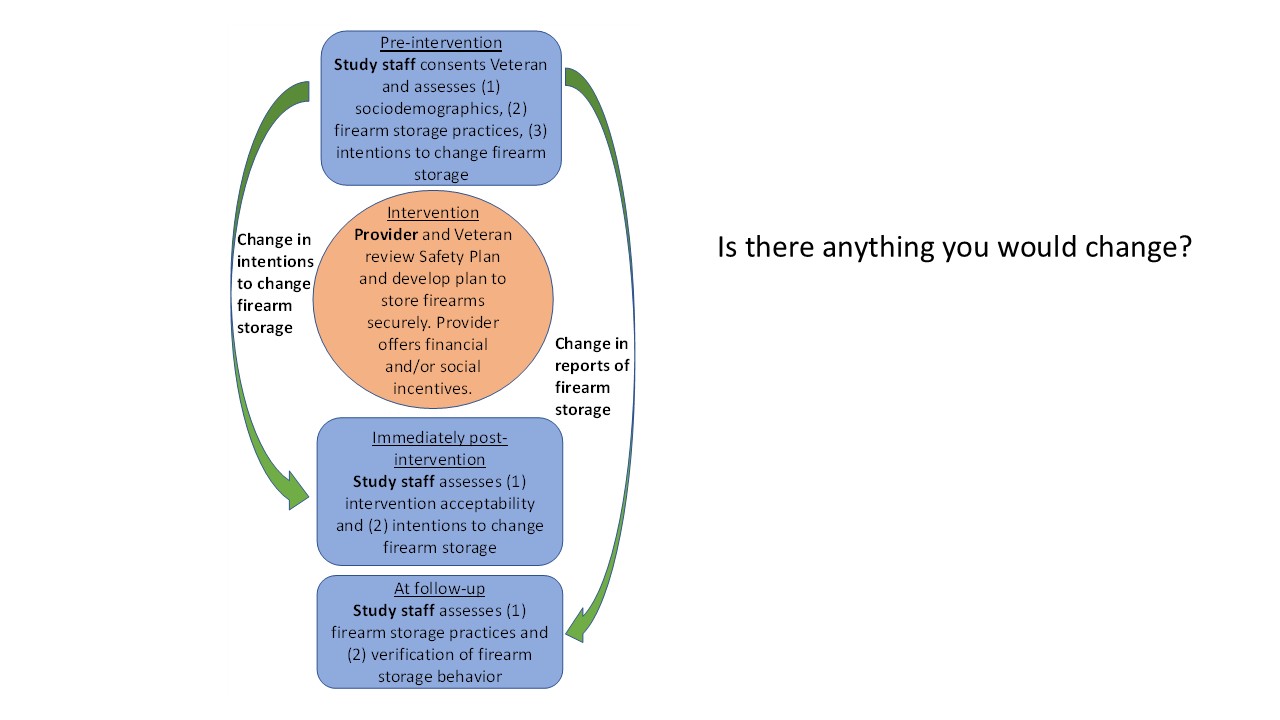
**

**Supplemental Item 1. Interview Guide (Veterans)**

*Thank you for agreeing to participate in this study. Today I will ask you about your thoughts on a new suicide prevention program we are developing. We hope to use the information you share with us to improve the services we offer Veterans at risk for suicide.*

***Please remember that there are no right or wrong answers for today’s interview, and you should feel free to take your time answering our questions. You can skip any questions you do not want to answer, and may withdraw from the study at any time. Before we begin, I would like to confirm that you are in a secure and private location and that you are not doing an activity that may be distracting or harmful, like driving.***

***As we discussed, I will be audio recording the interview. Is it OK if I turn on the recording now?***

*We are doing this study because we know that firearms account for most suicides among Veterans. We also know that storing firearms more securely when Veterans are having thoughts of suicide can reduce the risk of injury or death for Veterans and their families. Storing firearms more* *securely includes storing firearms outside of the home, locking them up inside of the home, or locking up or storing parts of the firearm (like the firing pin) outside or inside of the home.*

*I will start by asking about your experiences discussing firearms with your providers. Please only share information about your firearm ownership that you feel comfortable discussing – our main goal is to get your general feedback about the intervention we’re studying.*

**[Clarifying reasons for owning firearms]**

1. What is the main reason you own firearms? How do you store them and why?

Probe: Is there a specific firearm that is more difficult to store securely (e.g., service firearm, firearm in your car, firearm you use for home protection)?

1. Have you ever talked to your VA provider about how you store your firearms? Can you walk me through your experience (how you felt about it)?

*Sometimes, Veterans can be reluctant to store their firearms more securely when they are having thoughts of suicide. We are considering developing a program where we encourage Veterans to store their firearms more securely by offering them a VCS gift card (which can be used in-person at any VA store, cafeteria, or coffee shop or online through their website) or another type of gift or debit card, or other types of help (for example, working with a Veteran’s family member or friend to help support the Veteran in making these changes).*

*I will now ask you some questions about this type of program. While this program may not apply to you, we are curious about your perspectives to help us understand how other Veterans might feel about it.*

1. In general, what do you think about the VA offering this type of program (i.e., offering gift cards or other help for secure storage)?

**[Offering financial and/or social incentives]**

**If possible, screen share with Veteran handout describing these options**

1. We are considering six different ways to encourage Veterans to store their firearms more securely as part of this program. We would like to hear your thoughts about each option – reasons you would or wouldn’t be interested in them, which ones you would prefer, and what would make you more or less likely to participate in the program if they were offered. These include:
   1. Offering Veterans a VCS or other gift card with a set amount of money for storing their firearms more securely (for example, $50 dollars).
   2. Offering Veterans the chance to win a larger VCS or other gift card for storing their firearms more securely (for example, including them in a lottery with a 25% chance of winning $200).
   3. Showing the Veteran a VCS or other gift card (for $50, for example) assuming that they will store their firearms more securely, and then not providing the gift card if the Veteran doesn’t make this change.

Probe: For the monetary incentives, ask Veterans how interested they would be in the incentive, if they have any concerns about it, and what would make them more or less likely to participate in the program if it were offered.

- 1. Providers working with Veterans to choose a family member or friend they trust to help the Veteran store their firearms more securely (for example, helping the Veteran buy a lock box or find a storage facility).
  2. Providers sharing real examples of other Veterans who decided to store their firearms more securely because they had concerns about keeping themselves safe from suicide.
  3. Providers asking Veterans to write down their reasons for storing their firearms more securely to benefit other Veterans in the future.

Probe: For the social incentives, ask Veterans how interested they would be in the incentive, if they have any concerns about it, and what would make them more or less likely to participate in the program if it were offered.

- 1. Ask Veteran to rate [1] their top monetary incentive option (A-C), [2] their top social incentive option (D-F), and [3] also whether they prefer monetary vs. social.

1. What amount of money would a VCS gift card need to be to motivate you to store your firearms more securely?
2. Are there any other rewards that Veterans would be more interested in getting rather than a VCS gift card, like a gift card to another store or cash to use however they wanted?

**[Verifying changes in firearm storage]**

**If possible, screen share with Veteran handout describing these options:**

1. There are also several options for Veterans to show providers that they changed the way they store firearms in order to receive a VCS or other gift card. We would like to hear your thoughts about each option – reasons you would or wouldn’t be interested in them, which ones you would prefer, and what would make you more or less likely to participate in the program if you were asked to use them. They include:
   1. Showing or sending providers (1) a picture of the storage device or storage facility, or (2) the receipt from a recently purchased device or storage space in a facility (for example, a picture or receipt of the safe or lock that the Veteran bought, or the place the Veteran brought their firearms).

Probe: For each option, ask Veterans what they think about the option, if they have any concerns about it, and what would make them more or less likely to participate in the program if they were asked to use it.

- 1. Showing or sending providers a picture of the securely stored firearm.
  2. Among these options, what is your top choice?
  3. Using MyHealthEVet to send providers this information, sending it through a smartphone app, an encrypted email, or using a telehealth platform to show providers this information (e.g., VA Video Connect), or providing this information another way.

Probe: Among these options, what is your top choice? (have Veteran choose the best among the listed options even if they bring up another option)

1. If a Veteran owns multiple firearms, what firearm should this program focus on? (If Veteran does not understand question, prompt by asking – the least secured firearm, some other firearm…)
2. What are your thoughts about how providers document these conversations about firearm storage in Veterans’ medical records?

**[Introducing and framing the add-on intervention]**

1. Which member of your treatment team would it be easiest to discuss this program with (e.g., psychiatrist, psychologist, team social worker or nurse, someone else)?
2. How can providers introduce this program with Veterans to encourage them to participate in it?

Probe: What are other ways of encouraging Veterans to participate in this program aside from having their providers discuss it with them (e.g., flyers)?

1. What method of storing firearms do you think we should encourage Veterans at risk of suicide to use? Examples include storing firearms outside of the home, storing firearms inside the home without access to the keys or combination lock, or storing firearms at home locked and separately from ammunition.

**[Following up to encourage continued behavior change]**

1. How many times, and for how long, should providers follow up with Veterans after their initial conversation to encourage them to store their firearms more securely?

Probe: Should Veterans be offered additional gift cards for continuing to store their firearms more securely for longer periods of time?

Probe: Is there any other way to encourage continued secure storage of firearms?

**[Role of sociodemographic characteristics on perceptions of the add-on intervention]**

1. There can be characteristics about people’s backgrounds (for example, their culture, community, race/ethnicity, gender, or parenting status) that impacts the way they feel about firearms, firearm storage, and the type of program we’ve been talking about. How does your background impact your feelings about these topics?

**[Individualizing the add-on intervention]**

1. How do you think we could adapt this program to best meet your or other Veterans’ needs or concerns?

Probe: Can you think of other strategies we haven’t discussed to make it more convenient or appealing?

1. Do you have any other thoughts or concerns about this type of program you want to share with us?

Probe: Do you think other Veterans would have concerns about this type of program?

**Supplemental Item 2. Interview Guide (Clinicians and Administrators)**

*Thank you for agreeing to participate in this study. Today I will ask you about your thoughts on a new suicide prevention program we are developing. We hope to use the information you share with us to improve the services we offer Veterans at risk for suicide.*

***Please remember that there are no right or wrong answers for today’s interview, and you should feel free to take your time answering our questions. You can skip any questions you do not want to answer, and may withdraw from the study at any time.***

***I also want to highlight that participation in this interview is voluntary and that the information you share is confidential. Your anonymity will be protected in that your identity will be kept confidential by the research team and identifiers will be kept separate from the coded data. Results will be reported in aggregate and will therefore be anonymous in reports.***

***Before we begin, I would like to confirm that you are in a secure and private location and that you are not doing an activity that may be distracting or harmful, like driving.***

***As we discussed, I will be audio recording the interview. Is it OK if I turn on the recording now?***

*We are doing this study because as you may know, firearms account for most suicides among Veterans. As providers, talking to Veterans who are experiencing suicidal thoughts about storing their firearms more securely can reduce the risk of injury or death by suicide for Veterans and their families. Storing firearms more securely includes storing firearms outside of the home, locking them up inside of the home, or locking up or storing parts of the firearm (like the firing pin) outside or inside of the home.*

*I will start by asking about your experiences discussing firearms with your* *patients.*

**[Introduction]**

1. What is your role at the VA and how long have you worked here?
2. [Only clinicians] Can you walk me through your experiences discussing firearm storage with patients?

*Sometimes, Veterans can be reluctant to store their firearms more securely when they are having thoughts of suicide. We are considering developing a program where we encourage Veterans to store their firearms more securely by offering them a VCS gift card (which can be used in-person at any VA store, cafeteria, or coffee shop or online through their website) or another type of gift or debit card, or other types of help (for example, working with a Veteran’s family member or friend to help support the Veteran in making these changes).*

*I will now ask you some questions about this type of program.*

1. In general, what do you think about the VA offering this type of program?

**[Offering financial and/or social incentives]**

1. We are considering six different ways to encourage Veterans store their firearms more securely as part of this program. We would like to hear your thoughts about each option – reasons you would or wouldn’t be interested in these being offered to Veterans, if you have any concerns about them, and what would make it easier or harder to offer them to Veterans. They include:
   1. Offering Veterans a VCS or other gift card with a set amount of money for storing their firearms more securely (for example, $50 dollars).
   2. Offering Veterans the chance to win a larger VCS or other gift card for storing their firearms more securely (for example, including them in a lottery with a 25% chance of winning $200).
   3. Showing the Veteran a VCS or other gift card (for $50, for example) assuming that they will store their firearms more securely, and then not providing the gift card if the Veteran doesn’t make this change.
   4. Providers working with Veterans to choose a family member or friend they trust to help the Veteran store their firearms more securely (for example, helping the Veteran buy a lock box or find a storage facility).
   5. Providers sharing real examples of other Veterans who decided to store their firearms more securely because they had concerns about keeping themselves safe from suicide.
   6. Providers asking Veterans to write down their reasons for storing their firearms more securely to benefit other Veterans in the future.
   7. Among these options, what are your top 2? Why? [Specifically ask participant to rate their top monetary incentive option (A-C) and their top social incentive option (D-F)]
   8. When in the process of discussing firearm storage with Veterans would it make the most sense to offer a gift card or the other types of encouragement we just mentioned?
2. [Only for administrators] How much money, per Veteran, do you think the VA would dedicate to providing Veterans with gift cards as part of this program?

**[Verifying changes in firearm storage]**

1. There are also several options for Veterans to show providers that they changed the way they store firearms in order to receive a VCS or other gift card. We would like to hear your thoughts about each option – reasons you would or wouldn’t want to ask Veterans to use them, if you have any concerns about them, and what would make it more or less likely that Veterans or providers would want to use them. They include:

1. Showing or sending providers a picture of the storage device or storage facility, or the receipt from a recently purchased device or storage space in a facility (for example, a picture or receipt of the safe or lock that the Veteran bought, or the place the Veteran brought their firearms).
2. Showing or sending providers a picture of the securely stored firearm.
3. Among these options, what is your top choice?
4. Using MyHealthEVet to send providers this information, sending it through a smartphone app, an encrypted email, or using a telehealth platform to show providers this information (e.g., VA Video Connect), or providing this information in another way.

Probe: Among these options, what is your top choice?

1. If a Veteran owns multiple firearms, what firearm should this program focus on (all of them or only the least secured firearm, like the one by their bedside)?

**[Introducing and framing the add-on intervention]**

1. Which member of a Veteran’s treatment team (e.g., psychiatrists, psychologist, team social worker or nurse, someone else) should offer Veterans gift cards or other encouragement to integrate this program best into clinic flow?

Probe: Is there anything else we can do to help integrate this program into clinic flow?

Probe: How do you think we should handle suicide risk that comes up if someone other than the Veteran’s main mental health provider offers the Veteran gift cards or other encouragement for secure storage?

1. How could we encourage providers to offer this program to Veterans and also make it appealing to Veterans to participate?

Probe: Is there any particular wording or framing you would use to introduce the program?

1. What method of storing firearms do you think we should incentivize Veterans to use (e.g., storing firearms outside of the home, storing firearms inside the home without access to the keys or combination lock, or storing firearms at home locked and separately from ammunition)?

**[Following up to encourage continued behavior change]**

1. How many times, and for how long, would you suggest that providers follow up with Veterans to see if they made changes to their storage of firearms?

Probe: Should Veterans be offered additional gift cards for continuing to store their firearms more securely for longer periods of time? Is there any other way to encourage continued secure storage of firearms?

**[Individualizing the intervention]**

1. How do you think we could adapt this program to best meet Veterans’ backgrounds and needs (e.g., rural versus urban location, race/ethnicity, gender, reasons for owning firearms)?

Probe: Can you think of any other strategies to make the program more convenient or appealing to Veterans or providers?

1. Do you have any other thoughts or concerns about this type of program that you want to share with us?
